# The inability of a dengue NS1 ELISA to detect Zika infections

**DOI:** 10.1038/s41598-019-55160-4

**Published:** 2019-12-09

**Authors:** Monique da Rocha Queiroz Lima, Thais Chouin-Carneiro, Elzinandes Azeredo, Luciana Santos Barbosa, Thiara Manuele Alves Souza, Jéssica Badolato Corrêa da Silva, Priscila Conrado Guerra Nunes, Márcia Dal Fabbro, Izilyanne H. Romanholi Facco, Rivaldo Venâncio-da-Cunha, Flavia Barreto dos Santos

**Affiliations:** 1 Viral Immunology Laboratory, Oswaldo Cruz Institute, Rio de Janeiro, 21045-360 Brazil; 2 Medical Clinic Department, Federal University of Mato Grosso do Sul, Campo Grande, 79070-900 Brazil

**Keywords:** Infectious-disease diagnostics, Viral infection

## Abstract

The presence of dengue virus (DENV), Zika virus (ZIKV) and Chikungunya virus (CHIKV) in Brazil, may result in a difficult diagnosis due to the signs and symptoms shared by those. Moreover, as DENV and ZIKV belong to the same family, serological assays may show a high rate of cross-reactivity. Here, we evaluated a Dengue NS1 capture assay for early and differential diagnosis of dengue during the Zika epidemic occurred in Brazil in 2016. Samples (n = 227) from 218 patients included sera, plasma and urine from previously confirmed acute cases of Zika, dengue and Zika/dengue co-infections. Nine of those patients presented two specimens. The Dengue NS1 test was very specific for dengue diagnosis (99.32%), even in the co-circulation with ZIKV, and exhibited a high accuracy in not detecting acute Zika infections (92.43%). Our findings showed that the dengue NS1 capture test analyzed here was not able to recognize the ZIKV NS1 and its potential for cross-reaction.

## Introduction

Dengue virus (DENV) and Zika virus (ZIKV) belong to genus *Flavivirus* and are of a relevant impact for the public health. *Aedes* mosquitoes are the major vectors and are responsible for those arboviruses’ transmission in the tropical and subtropical regions of the world^1^. DENV infections can be caused by any of the four antigenically distinct serotypes (DENV 1 to 4) and can range from a nonspecific febrile illness to a more severe disease, characterized by thrombocytopenia, increased transaminases levels and plasma leakage, which may result in complications and death^2^. ZIKV infected patients, typically present symptoms such as fever, rash, arthralgia, myalgia, fatigue, headache and conjunctival hyperemia^3,4^. Despite the Zika-related congenital syndrome that may affect fetuses and babies, an infected person usually recovers completely, and fatalities are rare^5^.

DENV/ZIKV co-infections may occur where those viruses co-circulate, however the impact of those in the disease severity, is not fully known and requires further investigations. Some studies have reported arboviruses co-infected patients recovering after a mild clinical course of the disease^6–8^, but some may evolve with severe neurological manifestations^9^. Nevertheless, dengue differentiation from other arboviruses is crucial in endemic areas since an early diagnosis may allow the monitoring of potential markers for dengue severity.

In general, the signs and symptoms caused by those arboviruses are very similar and may be troublesome for differential diagnosis and patient management^10^. Due to the difficulties in clinically diagnosing those infections, the laboratory plays an important role. However, the tests should have maximum sensitivity, specificity and be simple, to provide an early support to patients by accurately differentiating dengue from Zika and other febrile diseases^11,12^. Mainly, the laboratorial diagnosis of dengue and Zika relies on the molecular detection of the virus^11–13^, however, a negative result does not exclude infection due to the low virus titer depending on the sample collection timeframe. The ELISA (enzyme-linked immunosorbent assay) is still currently the simplest and most widely used diagnostic test^11^, however, there is still a lack of commercially available serological test, sensitive and specific enough, to distinguish both viruses^14,15^. Moreover, it has been shown that IgM, usually detected in most assays, is not considered a good confirmatory marker^12,14,16^.

Similarly to dengue, the ZIKV non-structural protein 1 (NS1) is involved in viral replication, immune evasion and pathogenesis^17,18^. Several NS1 ELISAs are commercially available for the early diagnosis of dengue, with good sensitivity and specificity^19–23^. Some studies have also demonstrated high sensitivity and limited cross-reactivity, suggesting that NS1 may represent an efficient differential assay between DENV and ZIKV infections^24,25^, as it has group-specific epitopes that potentially differentiates those viruses^12^. A dengue NS1 test cross-reacting with ZIKV infections would have significant consequences^26^. Here, we aimed to evaluate a dengue NS1 antigen capture assay for early and differential diagnosis of dengue during the Zika epidemic occurred in Brazil during 2016.

## Results

In this investigation, 227 samples from 218 suspected cases of arboviral infection, including serum, plasma and urine, were tested by molecular and serological methods. Arboviral infection was confirmed in 60.35% (137/227) of those cases, by of at least one of the laboratory diagnosis performed, and 39.64% (90/227) were negative.

Overall, ZIKV infection was confirmed in 25.11% (57/227) of the samples and DENV in 24.66% (56/227) by both molecular tests used, independently of the clinical specimen analyzed. ZIKV/DENV co-infections were identified in 10.57% (24/227) of the samples (DENV-1/ZIKV, n = 14, DENV-4/ZIKV, n = 8 and NS1-DENV/ZIKV, n = 2) Table 1. None of the samples tested were positive for CHIKV detection by real-time (rt) RT-PCR, nor anti-CHIKV IgM. All samples were also negative for MAYV detection using molecular diagnosis. The alphaviruses CHIKV and MAVY were investigated as differential diagnosis due to their occurrence and circulation in Brazil.

**Table 1:** Investigation of arboviral infections by molecular and serological methods during an outbreak occurred in Midwest Brazil, 2016.

Specimen	Molecular diagnosis			Serological diagnosis	
	rtRT-PCR for ZIKV (Lanciotti et al., 2008) Positive/Tested (%)	rtRT-PCR for DENV (Johnson et al., 2005) Positive/Tested (%) SEROTYPE	Simplexa™ Dengue rtRT-PCR Positive/Tested (%) SEROTYPE	Platelia Dengue NS1 Positive/Tested (%)	Anti-DENV IgM Capture Positive/Tested (%)
Serum (n = 76, all mono-infections)	33/76 (43.42)^a^	0/76^b^	0/76	5/76 (6.57)	0/76
Plasma (n = 132; 108 mono-infections and 24 co-infections)	12/132 (9.09)	22/132 (16.66)^c^ 14 DENV-1 (14/22; 63.63) 8 DENV-4 (8/22; 36.37)	22/132 (16.66)^c^ 14 DENV-1 (14/22; 63.63) 8 DENV-4 (8/22; 36.37)	58/132 (43.93)	29/132 (21.96)
Urine (n = 19, all mono-infections)	12/19 (63.15)	0/19	0/19	0/19	0/19
TOTAL	57/227 (25.11)	22/227 (9.69)	22/227 (9.69)	63/227 (27.75)	29/227 (12.77)

^a^All 33 positive serum samples by rtRT-PCR for ZIKV, were negative for Dengue (NS1 and IgM), chikununya and mayaro.

^b^From 76 dengue negative serum samples by rtRT-PCR, only 5 were positive for Dengue NS1. Those 5 samples were negative for Zika, chikungunya and mayaro.

^c^By either rtRT-PCR for DENV and Simplexa™ Dengue rtRT-PCR, 22 plasmas were positive for dengue and from those, 14 were characterized as DENV-1 and 8 as DENV-4.

In ZIKV positive samples (n = 57), viral genome could be identified by rtRT-PCR in serum (43.42% [33/76]), plasma (9.09% [12/132]) and urine (63.15% [12/19]). Molecular detection of DENV was possible in 9.69% (22/227) of the samples tested by either rtRT-PCR and/or Simplexa™ Dengue real-time RT-PCR and were all in the plasma. By using both tests, DENV-1 was the infecting serotype in 63.63% (14/22) of the positive samples and DENV-4 in 36.37% (8/22). None of the serum and urine samples were positive for dengue by the molecular assays used, Table 1.

Anti-DENV NS1 was identified in 27.75% (63/227) of the samples analyzed. A higher positivity was observed in the plasma (43.93%, 58/132). Five out of 76 serum samples (6.57%) were also positive, but no positivity was observed in the urine samples. Anti-DENV IgM antibodies were detected only in the plasma and in 29 samples (21.96%, 29/132), as assessed by MAC-ELISA, Table 1.

The analysis showed that 10.71% (6/56) of DENV mono-infected patients were also anti-DENV IgM positive. On the DENV/ZIKV co-infected patients, anti-DENV IgM was detected in 8.33% (02/24) of the cases. Two ZIKV mono-infected patients (3.50%; 02/57) were also anti-DENV IgM positive, but had a negative result by RT-PCR for DENV. One negative case (0.73%; 01/137) for DENV and ZIKV by RT-PCR was also negative for the Dengue NS1 test, however it presented a positive result for anti-DENV IgM and anti-CHIKV IgM.

In this analysis, a high sensitivity of the Platelia NS1 test (79.74%) was observed in confirming dengue cases. Based on the analysis of all clinical samples (serum, plasma and urine), the test showed a specificity of 99.32%. The test showed a high accuracy in the non-detection of acute ZIKV infections (92.51%), with a positive predictive value of 98.43% and negative predictive value of 90.18%, Table 2.

**Table 2:** Sensitivity, specificity, accuracy, predictive values and likelihood ratio values of the commercial dengue NS1 capture assay used for dengue diagnosis.

Statistics* (Platelia Dengue NS1 Ag ELISA)	(%)
Sensitivity (63/79)	79.74
Specificity (147/148)	99.32
Accuracy (210/227)	92.51
Positive Predictive Value (63/64)	98.43
Negative Predictive Value (147/163)	90.18
Likelihood Ratio of a Negative Test Result	0,21
Likelihood Ratio of a Positive Test Result	79

*Sensitivity (a/a + b), specificity (d/c + d), accuracy (a + d/a + b + c + d), positive predictive value (a/a + c), negative predictive value (d/b + d), likelihood ratio of a negative test result (sensitivity/1-specificity) and likelihood ratio of a positive test result (1-sensitivity/specificity), where a = true positive (n = 63), b = false negative (n = 16), c = false positive (n = 1) and d = true negative (n = 147).

The use of the Dengue NS1 test showed no positive results when ZIKV cases were tested, but one case presented an inconclusive result. Of all cases, six presented serum and urine samples, four of those, were from Zika cases and dengue NS1 negative, and two were negative. Only one dengue case presented plasma and serum, but was negative in the Dengue NS1 test.

**Figure 1:**
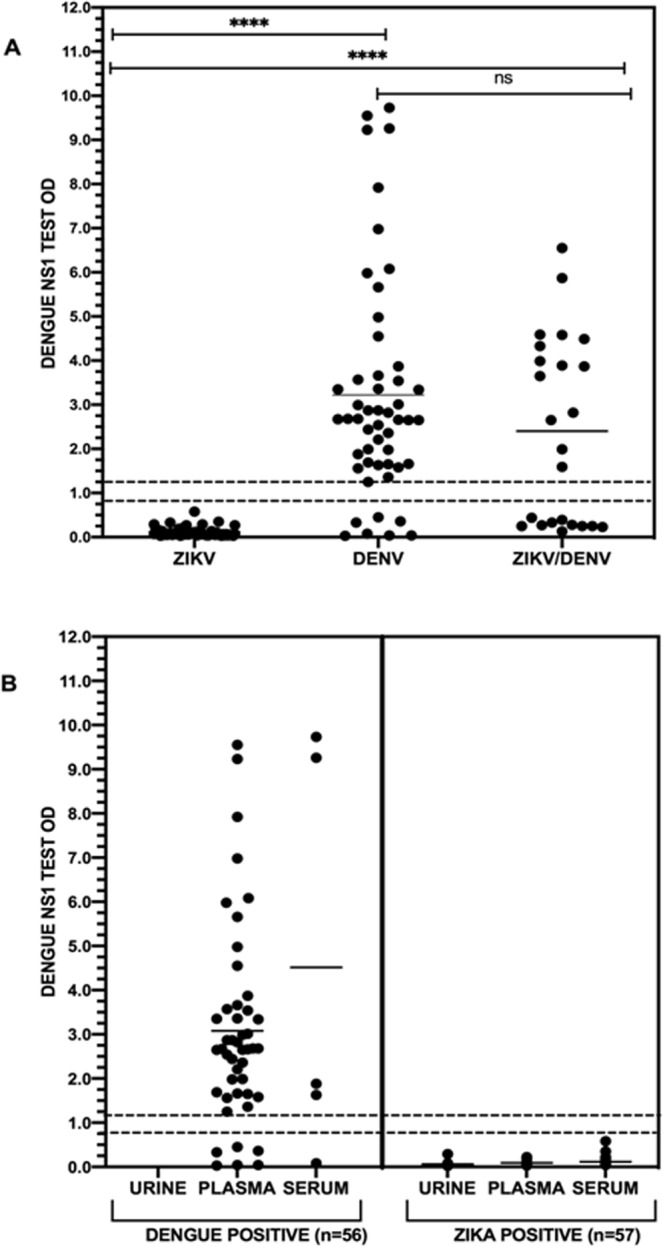
Dengue NS1 capture ELISA absorbance on Zika and dengue mono and co-infections (**A**) and according to distinct specimens from Zika and dengue positive cases (**B**). The nonparametric Mann–Whitney U test was used to evaluate differences between optical density (OD) among DENV and ZIKV mono and co-infections. ****p < 0.0001, ns: not significant, (—) represents the mean value for each group and (---) dashed lines, the cut-off interval for the test.

It is suggested that the dengue NS1 test is very specific for dengue, without presenting cross-reactivity with ZIKV positive samples. In fact, DENV positive cases were significantly more likely to be positive in the Dengue NS1 antigen capture test than the ZIKV positive ones (49/56 versus 0/57, p = 0.000). Likewise, samples co-infected with DENV/ZIKV were significantly more likely to be positive for the NS1 test than the ZIKV mono-infected ones (14/24 versus 0/57, p = 0.000), Table 3. Moreover, the NS1 optical densities (OD) were significantly higher in DENV mono-infections when compared to those from the ZIKV mono-infections (p < 0.0001), even when those were compared to the co-infected ZIKV/DENV group (p < 0.0001). The OD values obtained in the distinct groups and in the distinct specimens are shown on the Fig. 1.

**Table 3:** Sensitivity of the dengue NS1 capture assay in Zika and dengue, mono-infections and co-infections.

Type of arboviral infection	Platelia Dengue NS1 Ag-ELISA		
	Positive (%)	Inconclusive (%)	p value
DENV mono-infection (n = 56)	49/56 (87.5)	—	0.000 (DENV vs ZIKV)
ZIKV mono-infection (n = 57)	0/57	1/57 (1.7)	
DENV/ZIKV co-infections (n = 24)	14/24 (58.33)	—	0.000 (DENV/ZIKV vs ZIKV)

## Discussion

Dengue is a major public health problem in the tropical and subtropical regions of the world, making the rapid diagnosis crucial to limit the spread of the disease^11^. However, with the ZIKV introduction in many regions, the real incidence of infection was unclear, due to the high rates of cross-reactivity among those arboviruses^13^. The infection is transient and mild, but is strongly neurotropic and may cause teratogenic effects in fetuses of infected mothers^18,27^.

ZIKV and DENV are not only transmitted by the same vector^28^ and have similar signs and symptoms, but also share structural similarities, as they belong to the same family. Therefore, their induction of highly cross-reactive antibody responses^29–31^ is troublesome for most serological approaches used for diagnosis. For dengue diagnosis, the ELISA remains the most widely used diagnostic test and, besides its use to detect anti-DENV IgM antibodies, the assay is also used to capture DENV NS1 glycoprotein produced by infected host cells. Moreover, it has been shown that ELISAs are inexpensive and sensitive enough to detect analytes present at very low concentrations^11,32^. However, the analysis of an anti-dengue IgM test indicated that ZIKV infected patients may present a positive result^33^. In that case, retesting by other diagnostic techniques is necessary to provide a more reliable result and, the use of virus isolation and molecular methods is useful. Here, the use of the rtRT-PCR, was imperative in the confirmation of ZIKV and DENV mono and co-infections, and should be a strategy for laboratory test algorithms.

DENV NS1 is a unique diagnostic marker for the early diagnosis of dengue compared to other serological tests, such as the MAC-ELISA, because it is detected in patients’ serum shortly after the onset of symptoms^12,19^. Herein, the Platelia NS1 test presented a high sensitivity and specificity in confirming dengue cases based on the analysis of all clinical samples (serum, plasma and urine) and a high accuracy in the non-detection of acute ZIKV infections (92.51%).

For Zika, most diagnosis relies on the use of rtRT PCR and MAC-ELISA in urine, serum, plasma or cerebrospinal fluid^13,29,34^, however, due to cross-reactivity with DENV^35^, the confirmation rate of presumptive positive results may be less than 50% for some commercially available Zika IgM ELISA assays^36^.

The ZIKV NS1 induces a virus-specific non-neutralizing antibody response and it represents a reliable diagnostic target^24^. Moreover, it has been shown that most anti- ZIKV NS1 antibodies from patients with primary infection are specific to ZIKV, but more than 50% of those with a prior DENV exposure, react to dengue^37^. In fact, cross reactivity in human anti-NS1 antibodies increases after sequential DENV and ZIKV infections^24^. Despite that, an ELISA using ZIKV NS1 was able to differentiate Zika and dengue^14,33^. Recently, the combined use of the four DENV and ZIKV NS1 proteins in an ELISA format was able to distinguish DENV from ZIKV infections^38^.

Our findings suggest the inability of the Dengue NS1 capture test to recognize the ZIKV NS1 in different specimens used for both dengue and Zika diagnosis, and this information is important when choosing a reliable test for epidemiological surveillance and differential diagnosis between those infections. Despite that, wider evaluations in distinct settings are also welcome and needed to corroborate those observations.

## Methods

The specimens analyzed in this study were from an ongoing Project approved by the Oswaldo Cruz Foundation Ethic Committee (CAAE 57221416.0.1001.5248). All patients enrolled signed an informed written consent. An infectious disease physician collected the patients’ data using a structured questionnaire and the patient’s personal information was anonymized before the data was accessed. This cross-sectional and observational study was carried out at the Healthy Unit UPA Coronel Antonino in Campo Grande, MS, Brazil from February to March of 2016. The patients’ clinical and laboratory characteristics were published elsewhere^8^. All arboviral suspected cases were obtained during an active surveillance performed by the Laboratory of Viral Immunology, IOC/FIOCRUZ, Rio de Janeiro, Brazil and all laboratorial methods were performed in accordance with relevant guidelines and regulations.

### Laboratorial diagnosis

Clinical samples (n = 227; 76 sera, 132 plasma and 19 urine samples) from arboviruses suspected cases (n = 218) were screened by molecular and serological tests for DENV, ZIKV and CHIKV, as differential diagnosis. Nine of those cases presented 2 distinct specimens. Briefly, for the serological diagnosis of dengue, the Dengue Virus IgM Capture DxSelect™ (Focus Diagnostics, California, USA), and Platelia^™^ Dengue NS1 Ag ELISA (BioRad Laboratories, California, USA) tests were used. The Platelia^™^ Dengue NS1 Ag ELISA test analyzed is based on a one-step sandwich format microplate enzyme immunoassay to detect DENV NS1 antigen. The test uses murine monoclonal antibody for capture and revelation. If NS1 antigen is present in the sample, an immune-complex monoclonal antibody – NS1- monoclonal antibody/peroxidase will be formed. Briefly, the specimens were allowed to thaw to laboratory ambient temperature (21–22 °C). Sample diluent (50 µl), samples and controls (50 µl each) and 100 µl of diluted conjugate were incubated for 90 min at 37 °C within the respective microplate wells coated with purified mouse anti- NS1 monospecific antibodies. After a six-times washing step, 160 µl of substrate was added into each well and incubated for 30 min at room temperature in the dark. The presence of immune-complex was demonstrated by a color development and the enzymatic reaction was stopped by the addition of 100 µl of 1 N H_2_SO_4_. The optical density (OD) reading was taken with a spectrophotometer at a wavelength of 450–620 nm and the amount of NS1 antigen present was determined by comparing the OD of the sample to the OD of the cut-off control.

DENV molecular detection and serotyping were performed by conventional RT-PCR^39^ and by rtRT-PCR^40^ as described previously. Aiming to further exclude DENV infection in false negative cases, all samples were tested by using the Simplexa™ Dengue Real Time RT-PCR (Focus Diagnostics, California, USA) according to the manufacturer´s protocol, for DENV qualitative detection and typing. Due to the flaviviruses cross-reactivity in serological assays, for Zika investigation, cases were tested by One-step rtRT-PCR as described previously^29^. Chikungunya infection was assessed by using the rtRT-PCR protocol described elsewhere^41^. For the detection o anti-CHIKV IgM antibodies, the anti-CHIKV IgM ELISA (Euroimmun, Lubeck, Germany) was used according to the manufacturer’s protocol. All samples were also tested for Mayaro virus (MAYV) as previously described elsewhere^42^.

A Zika case was only considered, when positive by rtRT-PCR and negative for all other tests used for differential diagnosis. A dengue case was only considered when positive by a molecular test, accompanied or not, by a positive serological result (dengue NS1 and/or anti-DENV IgM) and negative for the other arboviruses tested. A chikungunya case was only considered when positive by rtRT-PCR, accompanied or not, by a positive serological result (anti-CHIKV IgM) and negative for the other arboviruses tested. ZIKV/DENV coinfections were considered when both viruses were detected simultaneously by rtRT-PCR specific for dengue and Zika, accompanied or not, by a positive serological result for dengue (dengue NS1 and/or anti-DENV IgM), as serology for Zika was not performed in this study. Negative cases were considered when samples presented a negative result in all tests performed here. After screening, confirmed Zika, dengue, ZIKV/DENV and negative cases were used for the analysis of the Platelia™ Dengue NS1 Ag-ELISA (BioRad Laboratories).

### Statistical analysis

Statistical analysis was performed using GraphPad Prism software, version 8.0 (GraphPad Software Inc., San Diego, California, USA). The nonparametric Mann–Whitney U test was used to evaluate differences between groups DENV and ZIKV. Values of p < 0.05 were considered significant for all statistical analysis.

## Data Availability

All data generated during this study are included in this article.
